# Anaerobic and Aerobic Performance of Elite Female and Male Snowboarders

**DOI:** 10.2478/v10078-012-0066-9

**Published:** 2012-10-23

**Authors:** Aleksandra Żebrowska, Dorota Żyła, Damian Kania, Józef Langfort

**Affiliations:** 1 Department of Physiology, Academy of Physical Education, Katowice, Poland.; 2 Department of Sports Training, Academy of Physical Education, Katowice, Poland.

**Keywords:** maximal oxygen uptake, anaerobic power, exercise training, snowboarding

## Abstract

The physiological adaptation to training is specific to the muscle activity, dominant energy system involved, muscle groups trained, as well as intensity and volume of training. Despite increasing popularity of snowboarding only little scientific data is available on the physiological characteristics of female and male competitive snowboarders. Therefore, the purpose of this study was to compare the aerobic capacity and maximal anaerobic power of elite Polish snowboarders with untrained subjects. Ten snowboarders and ten aged matched students of Physical Education performed two exercise tests. First, a 30-second Wingate test was conducted and next, a cycle ergometer exercise test with graded intensity. In the first test, peak anaerobic power, the total work, relative peak power and relative mean power were measured. During the second test, relative maximal oxygen uptake and lactate threshold were evaluated. There were no significant differences in absolute and relative maximal oxygen uptake between snowboarders and the control group. Mean maximal oxygen uptake and lactate threshold were significantly higher in men than in women. Significant differences were found between trained men and women regarding maximal power and relative maximal power. The elite snowboarders demonstrated a high level of anaerobic power. The level of relative peak power in trained women correlated negatively with maximal oxygen uptake. In conclusion, our results seem to indicate that the demanding competition program of elite snowboarders provides a significant training stimulus mainly for anaerobic power with minor changes in anaerobic performance.

## Introduction

Snowboarding is a new sports discipline, first included in the Olympic Games in 1998. Sub-disciplines have been developed as well as more demanding techniques, spectacular tricks, strength and the ability to generate high muscle power. Despite the increasing popularity of snowboarding over the last years, only few research projects investigated the physiological characteristics of competitive snowboarders. It has been documented that the physical performance of elite snowboarders depends on the type of discipline (half-pipe, giant slalom or snowboard cross). As each event requires specific physical attributes, the training effect might also vary with respect to the dominant energy system of the event, its duration, and number of races in each combination (Platzer et al., 2009; Dann et al., 2005; Hogg, 2003).

Recent studies have demonstrated that the percentage of fast twitch fibers, their requirement, and the ability to utilize the anaerobic energy system, although genetically determined, seem to be the most important physiological factors in competitive snowboarders (Raschner et al., 2009; Kipp, 1998). The short duration of half-pipe and other disciplines (10 to 30 s in duration) requires two general types of training, i.e., maximum speed and power exercises with repetitions up to 10 times and duration of 7–8 s and long rest periods, and 30 s runs repeated 3–5 times with incomplete recovery. These types of training increase maximal anaerobic power in response to higher concentrations of phosphocreatine kinase, greater activity of the enzymes for glycolysis and increase glycolytic synthesis of adenosine triphosphates. In addition to the glycolytic capacity of fast twitch fibers and potential functional changes in the motor nerves of involved muscle groups, the ability to sprint exercise is also improved (Bogdanis et al., 1996; Serresse et al., 1988; Prampero, 1981). Explosive and sustained anaerobic power has been shown to be important for competitive snowboarders. Greater leg power and increased hypertrophy of fast twitch muscle fibers may reduce fatigue, and consequently, the risk of snowboarders’ injuries (Bladin et al., 2004; Torjussen and Bahr, 2005).

Several studies have suggested that anaerobic power is an important factor for successful snowboarding performance. However, the benefits of aerobic training have also been documented (Platzer et al., 2009; Gross et al., 2009; Hogg, 2003). High aerobic physical efficiency increases the ability to recover from repeated bouts of anaerobic exercise; it could also increase muscle endurance and allow to tolerate greater training loads as well as reduce the risk of overtraining (Neumayer et al., 2003). Training for aerobic power and endurance stimulates the activation of aerobic metabolism (training at VO_2max_) (Bangsbo et al., 2000; Coyle, 1995). The exercise intensity should be below the anaerobic threshold (AT) and adequately adapted to AT limitations during the annual training cycle. As muscle fiber composition is genetically determined, exercise-induced increase in anaerobic efficiency is much less responsive to training than aerobic power (Bangsbo, 1996; Basset and Howley, 2000). However, these beneficial effects can be diminished if athletes became engaged in aerobic training (Noakes, 2000; Booth et al., 1998).

In their recent study, Platzer et al. (2009) described the physiological characteristics of Austrian national team snowboarders and concluded that maximal aerobic power is a good predictor for snowboard performance. Interestingly, a positive correlation between the physiological factors of aerobic capacity and FIS (Federation Internationale de Ski) scores and World Cup points was observed only in female competitors. The authors speculated that, in snowboarding, women’s performance levels were reflected in the test results more than those of men, probably because fitness, not technique, played an essential role in women’s events. Therefore, our hypothesis was that aerobic and anaerobic competition training might induce different physiological responses in elite snowboarders. To test this hypothesis, we compared the aerobic efficiency (VO_2max_ and AT) and maximal anaerobic power of elite Polish snowboarders with untrained subjects.

## Material and methods

Ten athletes (5 women -TrW, and 5 men -TrM), elite Polish snowboarders volunteered to participate in the experiment. Their training experience was 7.2 ± 1.2 years. All athletes underwent medical evaluations at the same time of the pre-season training including clinical history and physical examination. Five untrained women (CGW) and five men (CGM), aged matched students of Physical Education, served as control groups. Anthropometric characteristics of the study participants are presented in Table 1. The experimental procedures and possible risks were communicated verbally and in writing to all study participants, who then gave their informed written consent. The experiment was approved by the Ethics Committee of the Academy of Physical Education in Katowice and conformed to the standards set by the Declaration of Helsinki. The subjects were asked to abstain from strenuous exercise during three days before investigations. The subjects reported to the Laboratory after an overnight fast and performed two exercise tests. First, a 30-second Wingate test on a cycle ergometer (Monark, Sweden); the workload was individually calculated based on the following formula: workload = 0.075 x body mass (kg) (Bar-Or 1987). The exercise test was preceded by a 3-minute warm-up at 30 W. The test was started with maximal pedal rotations and discontinued after 30 seconds. During the test, peak anaerobic power (P_peak_), total work (W_max_), relative peak power (P_peak_/kg), relative mean power (P_mean_/kg), time to peak power (s), and anaerobic fatigue index (%) were registered.

The other test was conducted two hours after first one. The subjects performed a cycle ergometer exercise test with graded intensity, starting at 30 W, with 30 W increments every three minutes. Criteria for test termination were respiratory quotient greater than 1.1, heart rate of 180 beats per min., and/or physical exhaustion. Pulmonary ventilation (VE), oxygen uptake (VO_2_), and carbon dioxide output (CO_2_) were measured continuously from the 6^th^ min before starting the test, and throughout each stage of the exercise load using the Oxycon Apparatus (Jaeger, Germany). Heart rate (HR) was continuously recorded using the PE-3000 Sport-Tester (Polar Inc. Finland); systolic and diastolic blood pressures (SBP/DBP) were measured with a sphygmomanometer.

Blood samples were obtained from finger tip before and during the exercise (at one minute intervals) for determination of lactate concentration (LA). Blood LA was measured by an enzymatic method using commercial kits (Boehringer, Manheim, Germany). The lactate threshold (LT) was calculated according to Beaver et al. (1985).

All results are presented as the mean ± standard deviation. Significant differences in metabolic variables (LA, VO_2_, ExCO_2_, VE) and HR between examined groups were revealed with the Student’s t-test. To determine the inter-variable relationships between variables the Pearson correlation coefficients were analyzed. All the analyses were performed using the Statistica v. 9 Statistical Software package (StatSoft, Tulsa, OK, USA). Statistical significance was set at p < 0.05.

## Results

Mean age and body mass index were similar in female and male participants. The analysis of body composition demonstrated significant differences in body fat content (FM %) and fat free mass (FFM) between athletes and untrained subjects (Table 1). Significant differences in body height and composition (FM and FFM) were also found (Table 1).

### Aerobic power

**Figure 1:**
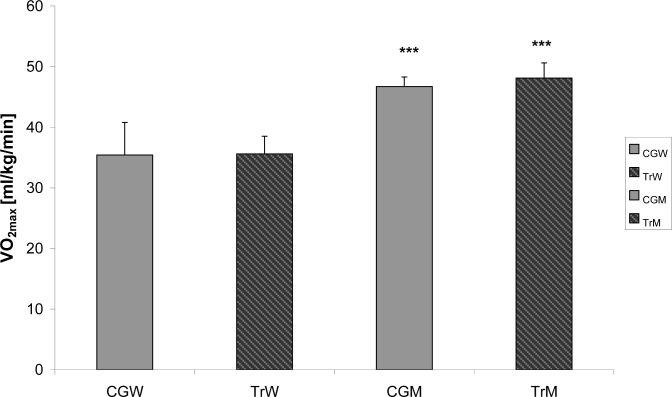
Maximal oxygen uptake in trained women (TrW) and men (TrM) compared to control participants: women (CGW) and male (CGM). Significant differences between women and men (***p<0.001)

Mean aerobic capacity measured at maximal intensity of graded cycle ergometer test did not differ significantly between athletes (both women and men) and untrained subjects (Figure 1).There were no significant differences in absolute and relative maximal oxygen uptake (VO_2max_) between snowboarders and the control. Interestingly, the maximal oxygen uptake measured in our elite snowboarders (TrW and TrM) was lower than VO_2max_ of female (50–55 ml/kg/min) and male (57–68 ml/kg/min) skiers (Willmore and Costill 2004).

The effects of gender on snowboarders’ aerobic power are summarized in Table 2.

Mean VO_2max_ was significantly higher in men (TrM) than in women (TrW) (p < 0.001). Significant differences were found between TrM and TrW regarding maximal power (P_peak_) (p < 0.01) and relative maximal power (P_peak_/kg) (p<0.05). No significant differences were observed in relative VO_2max_ to peak power (VO_2max_/P_max_), maximal heart rate (HR_max_), and HR measured at anaerobic threshold (HR_AT_). Maximal blood lactate concentration (LA) did not differ significantly between the two groups of athletes (p > 0.05). The lactate threshold (LT) occurred at a significantly higher power output in trained men compared to trained women (p < 0.01) (Table 2).

### Anaerobic power

**Figure 2:**
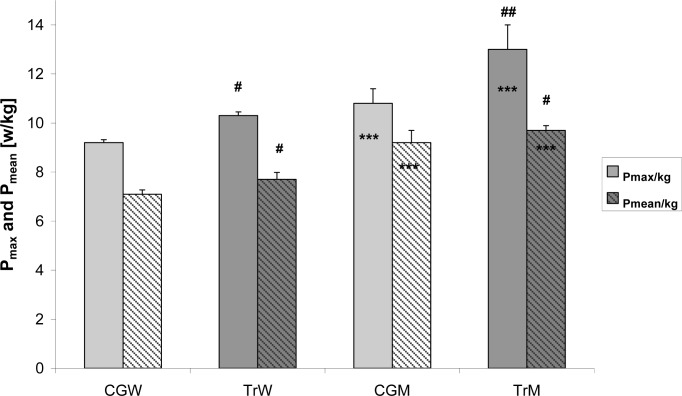
Maximal anaerobic power (P_max_) and mean anaerobic power (P_mean_) of trained women (TrW) and men (TrM) compared to control subgroups: female (CGW) and male (CGM). Significant differences between women and men (***p<0.001) and between trained and untrained subjects (# p<0.05; ## p<0.01)

Mean relative peak power (P_peak_/kg) and mean anaerobic power (P_mean_/kg) were significantly higher in elite female snowboarders compared to the control group (TrW *vs.* CGW; p<0.05) (Figure 2). Significant differences in P_peak_/kg and P_mean_/kg were also found between trained men and CGM (p < 0.01 and p < 0.05, respectively) (Figure 2). The elite snowboarders (TrM and TrW) demonstrated a high level of anaerobic power consistent with reference ranges for these populations (men > 10.2 W/kg; women > 9.8 W/kg).

Several indices of power output determined from a 30-second Wingate test in elite snowboarders are shown in Table 3. Mean peak anaerobic power (P_peak_) was significantly higher in trained men compared to trained women (p < 0.001) (Table 3). As expected relative peak power (P_peak_/kg) and mean anaerobic power (P_mean_/kg) were significantly greater in TrM than in TrW (p<0.001). In trained men no significant influence was observed of aerobic capacity on the variables of anaerobic power. High levels of relative peak power in trained women (TrW) correlated negatively with VO_2max_ (r = − 0.95 p < 0.05).

## Discussion

Wide ranges of laboratory tests have been used to optimize training loads for athletes (Carey and Richardson, 2003; Saltin and Ăstrand, 1967; Bouchard et al., 1991; Bangsbo, 1996; Bosco et al., 1982; Chimera et al., 2004; Higa et al., 2007; Coyle, 1995). However, there are limited data on the effects of physical training on aerobic and anaerobic capacity of elite snowboarders (Hogg, 2003; Platzer et al., 2009; Raschner et al., 2009). Our study examined the effect of training on maximal oxygen uptake and maximal anaerobic power of female and male snowboard competitors. The obtained results were compared to those of untrained controls. All female and male participants, both trained and untrained, did not differ with respect to age and body mass index (BMI) (Table 1). Therefore our results seem to confirm the effect of training on aerobic and anaerobic performance.

We found that elite snowboarders had significantly higher anaerobic power than age-matched controls. Relative maximal and mean anaerobic power were significantly greater in trained men compared to trained women (Figure 2). The progressive training program increased the competitive snowboarders’ ability to perform high intensity exercise in response to high anaerobic energy system involved. Moreover, a greater proportion of muscle mass, and probably, fast twitch mass and activation during exercise resulted in higher relative maximal anaerobic power in trained males compared to trained females (Hill and Smith, 1993; Zupan et al., 2009). Our observations are in agreement with previous data on the physiological characteristics of US Snowboard National Team suggesting that snowboarding events of short duration predominantly engage the ATP-PCr anaerobic energy system (Platzer et al., 2009; Kipp, 1998). Other disciplines, e.g., giant slalom, slalom, and snowboard cross (lasting approximately 30 seconds) requires a high glycolytic potential. Aerobic exercise is also included in the annual training cycle of world-class snowboarders to optimize their physiological performance. Aerobic power and endurance can improve during off-season, dryland, training, which should increase the muscle’s aerobic capacity and prepare snowboarders to maximize their physical performance during on-snow activities (Hogg, 2003; Neumayer et al., 2003). Most importantly, high aerobic power increases the ability to recover from repeated bouts of anaerobic power, and probably decreases lactate concentrations in response to higher LA utilization in slow twitch muscle fibers (Tesch, 1995; Gladden, 2000).

Despite a wide range of laboratory tests performed to determine the dominant energy system during muscle activities, little is known about physiological adaptations during snowboarding. Continuous heart rate (HR) monitoring during a snowboard run revealed an increase to 92% of the aged-predicted maximal HR suggesting the predominance of anaerobic processes. However, the average HR of 140 bts/min, seen in world-class snowboarders in the entire competition indicates that although the athletes use specific techniques with a predominance of anaerobic metabolism, a high aerobic capacity seems also important (Kipp, 1998).

Compared to untrained subjects, our trained female and male snowboarders demonstrated the same level of maximal oxygen uptake. The absence of significant differences in VO_2max_ between athletes and control subjects seems to indicate inappropriate aerobic training of the examined snowboarders. A low VO_2max_ confirmed low aerobic capacity of female and male snowboarders. Anaerobic threshold was also low, and amounted to 130W and 190W in female and male competitors, respectively. It should be emphasized that these values are characteristic of untrained populations (Bouchard et al., 1991; Carter et al., 2001).

Since anaerobic threshold is, at present, the most sensitive indicator of aerobic performance, the obtained values are suggestive of low cardiorespiratory adaptation to exercise and a decrease in aerobic energy production (Bangsbo et al., 2000; Basset and Howley, 2000; Whipp and al., 2007). Low aerobic capacity in response to repeated bouts of acute high-intensity exercise during the training process might act, at least partially, as a causative factor, decreasing the slow-to-fast twitch fiber ratio. These observations are consistent with our results suggesting a negative correlation between maximal anaerobic power and aerobic capacity in female snowboarders. In conclusion, our results seem to indicate that the demanding competition program of elite snowboarders provides a significant training stimulus for increasing and maintaining high anaerobic power and explosive strength. However, a problem remains of the competitors’ insufficient adaptation to disciplines of longer duration such as parallel slalom and parallel giant. Due to a potential negative effect of endurance training on anaerobic power, participation in aerobic exercise should be limited to the off-season and early preparatory periods of the annual training cycle.

## Figures and Tables

**Table 1:** Anthropometric characteristics of female and male elite snowboarders and the control group (Mean ± SD)

**Variable**	**Women (n=10)**		**Men (n=10)**	

	**Trained (TrW)**	**Control (CGW)**	**Trained (TrM)**	**Control (CGM)**
Age (y)	22.2 ± 5.1	21.5 ± 1.8	20.0 ± 0.7	21.2 ± 1.1
Body height (m)	168.8 ± 6.6	168.7 ± 4.1	179.8 ± 6.3*	175.4 ± 5.6
Body mass (kg)	60.3 ± 9.0	61.6 ± 8.3	71.8 ± 6.3	71.6 ± 5.6
BMI	21.4 ± 2.6	22.9 ± 2.5	22.2 ±1.5	23.2 ±0.7
FM (%)	14.9 ± 3.3	16.7 ± 1.6#	10.6 ± 3.2**	15.1 ± 0.7##
FFM (kg)	48.6 ± 6.4	51.4 ± 5.4#	63.0 ± 7.0*	60.7 ± 4.9**#**

Significant differences between women and men (*p<0.05; **p<0.01) and between trained and untrained subjects (# p<0.05)

**Table 2:** Variables of aerobic capacity measured using the bicycle ergometer test (Mean ± SD) Significant differences between women and men (*p<0.05; **p<0.01; ***p<0.001)

**Variable**	**Trained women (TrW)**	**Trained men (TrM)**
VO_2max_ (ml/min./kg)	35.6 ± 2.9	48.1 ± 2.5***
VO_2max_/P_max_ (ml/min./W)	9.8 ± 1.4	11.0 ± 1.1
P_max_ (W)	195 ± 30	286 ± 26.1**
P_max_ (W/kg)	3.7 ± 0.5	4.4 ± 0.4*
HRrest (bts/min)	71.0 ± 12.8	74.6 ± 4.9
HR_max_ (bts/min.)	182.8 ± 2.9	192.8 ± 8.8
SBP/DBP (mm Hg)	122 ± 17 /78 ± 8	130 ± 7/72 ±8
VE_max_ (L/min.)	85.1 ± 18.7	122.7 ± 12.6**
LArest (mmoL/L)	1.6 ± 0.3	1.9 ± 0.3
LA_max_ (mmoL/L)	11.9 ± 1.1	14.1 ± 2.0
P_AT_ (W)	130 ± 16.3	190 ± 16.3**
HR_AT_ (b/min.)	163 ± 12.7	168.8 ± 8.5

**Table 3:** Variables of anaerobic capacity measured by the Wingate Test (Mean ± SD)

**Variable**	**Trained women (TrW)**	**Trained men (TrM)**
P_max_ (W)	558.3 ± 87.6	899.0 ± 77.9***
P_max_ (W/kg)	10.3 ± 0.2	13.0 ± 1.0***
P_mean_ (W/kg)	7.7 ± 0.2	9.7 ± 0.2***
W_max_ (kJ)	12.9 ± 1.8	20.9 ± 1.6***

P_max_-maximal anaerobic power, P_mean_-mean anaerobic power, W_max_-total work.

Significant differences between women and men (***p<0.001)
